# Supplementary figures and images for: Sexual dimorphism in the complete connectome of the Drosophila male central nervous system
Source: bioRxiv. 2025 Oct 30:2025.10.09.680999. Preprint. [Version 2] doi: 10.1101/2025.10.09.680999 (PMC12636603; doi:10.1101/2025.10.09.680999)

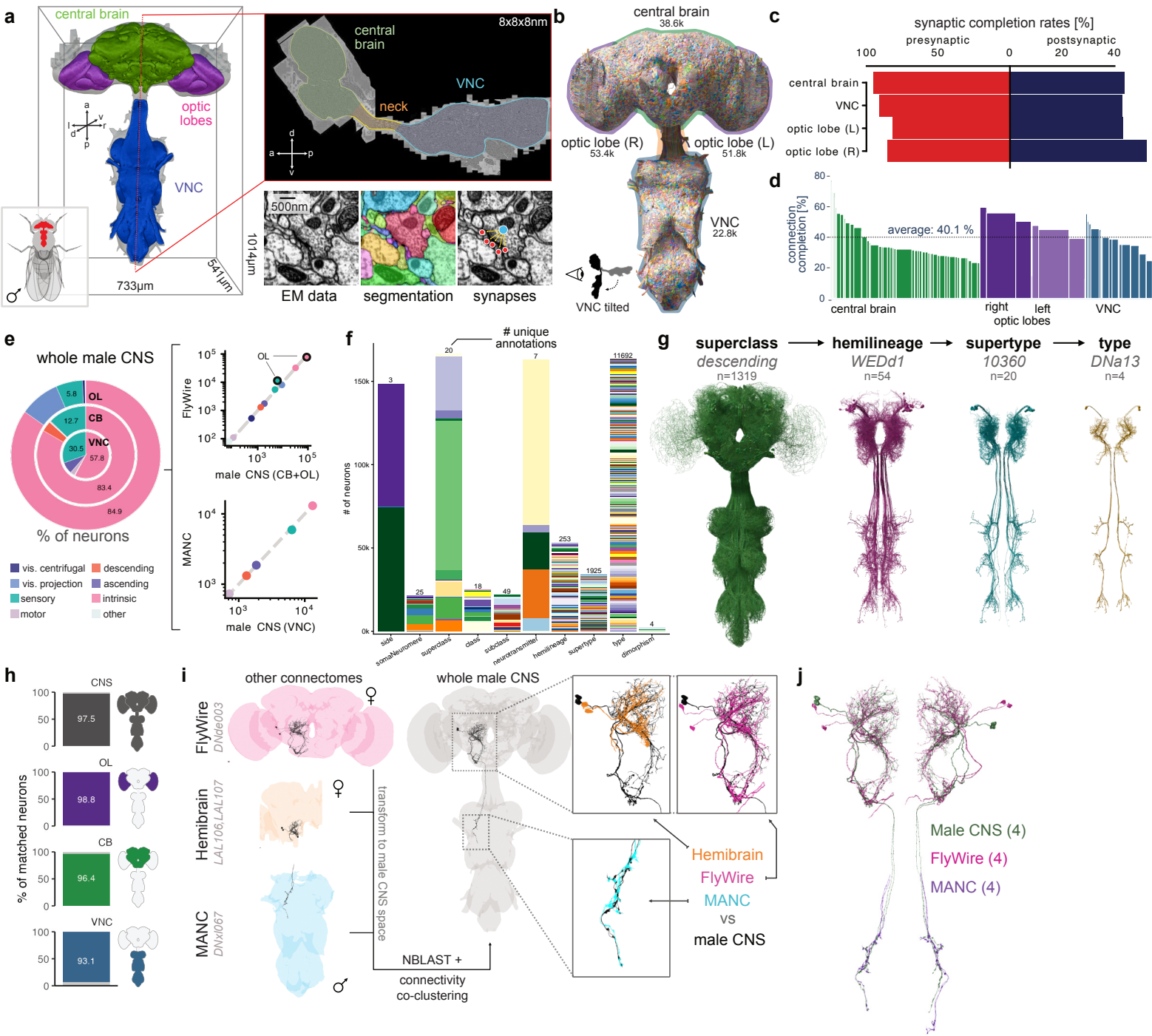

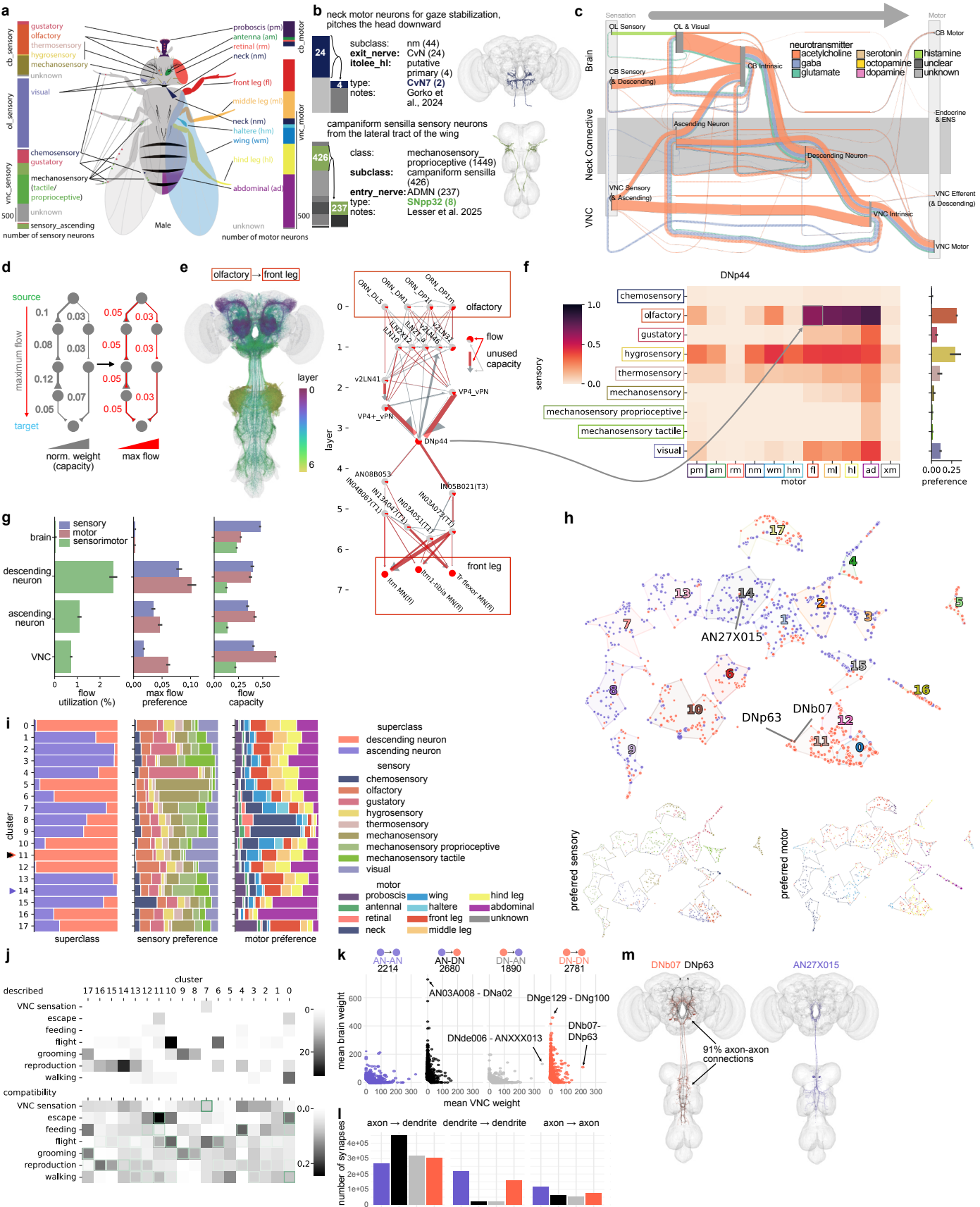

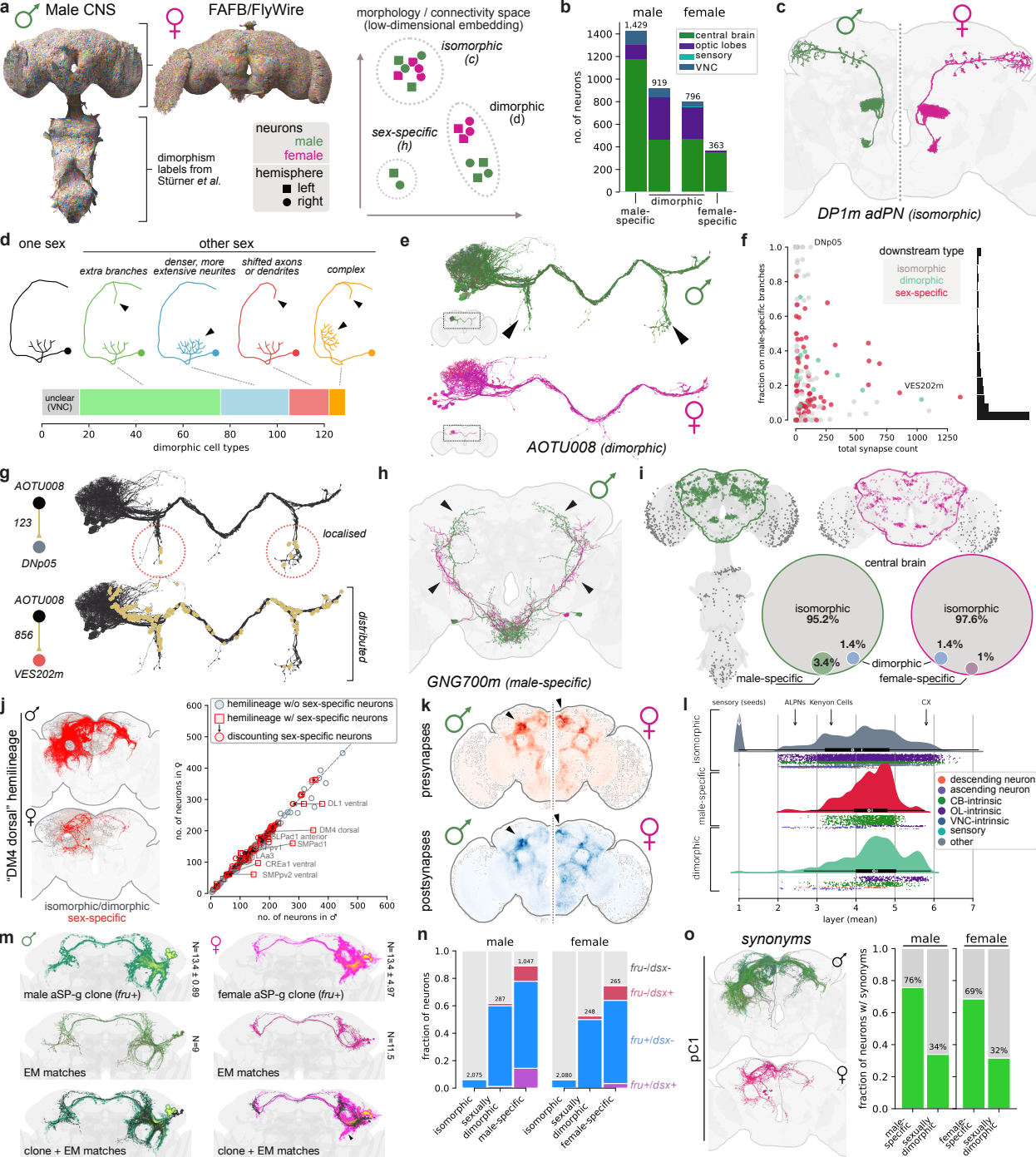

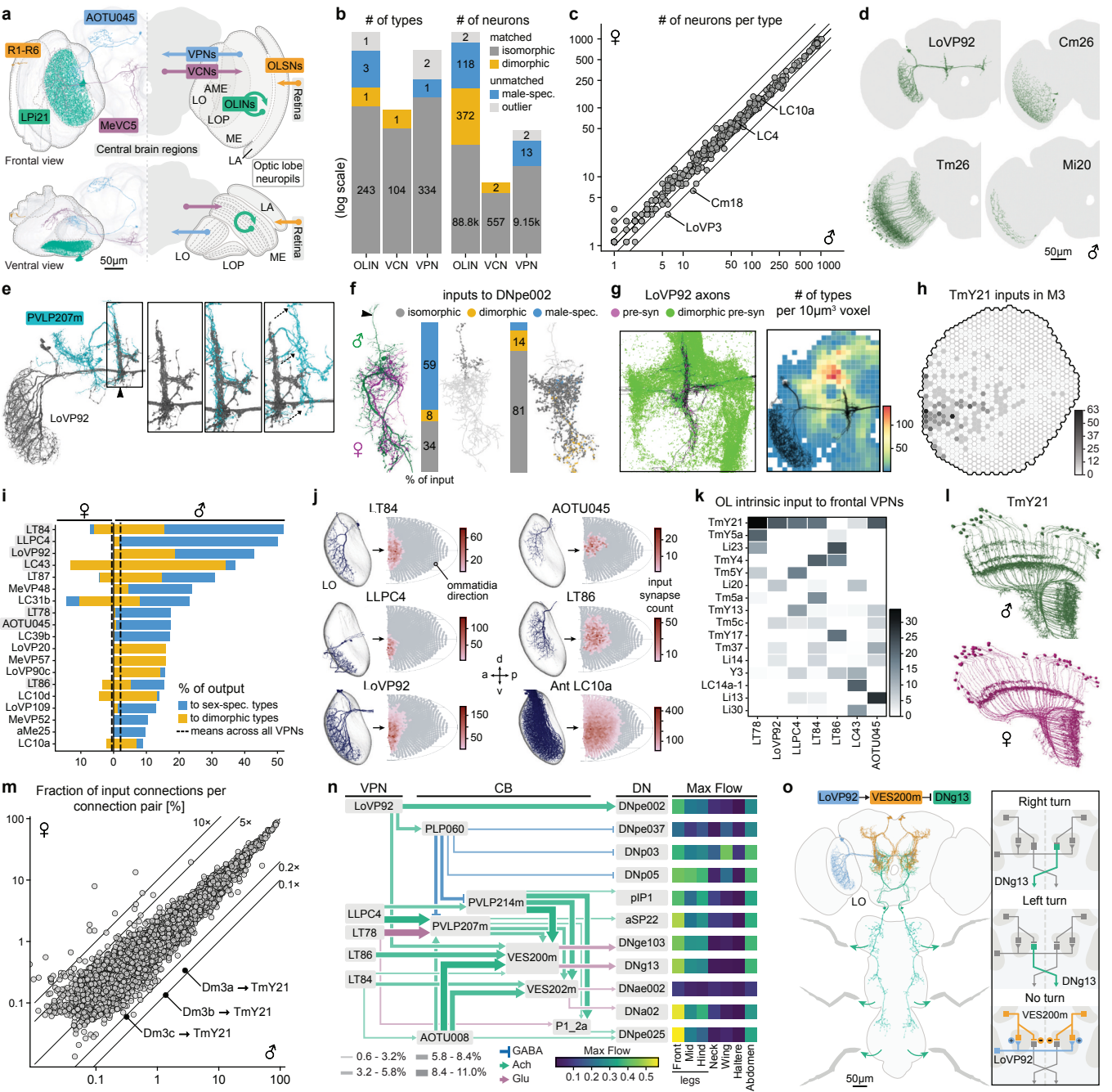

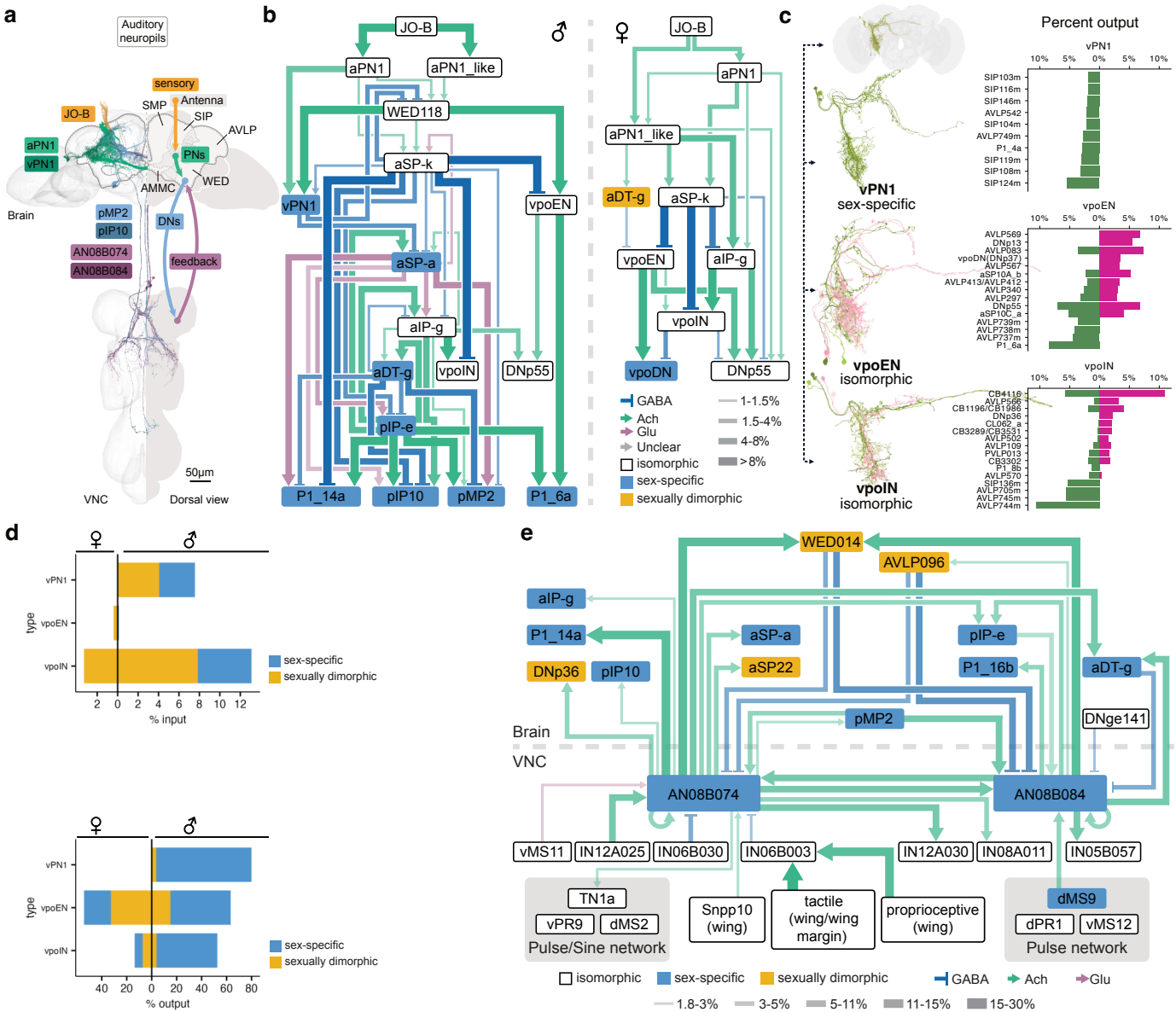

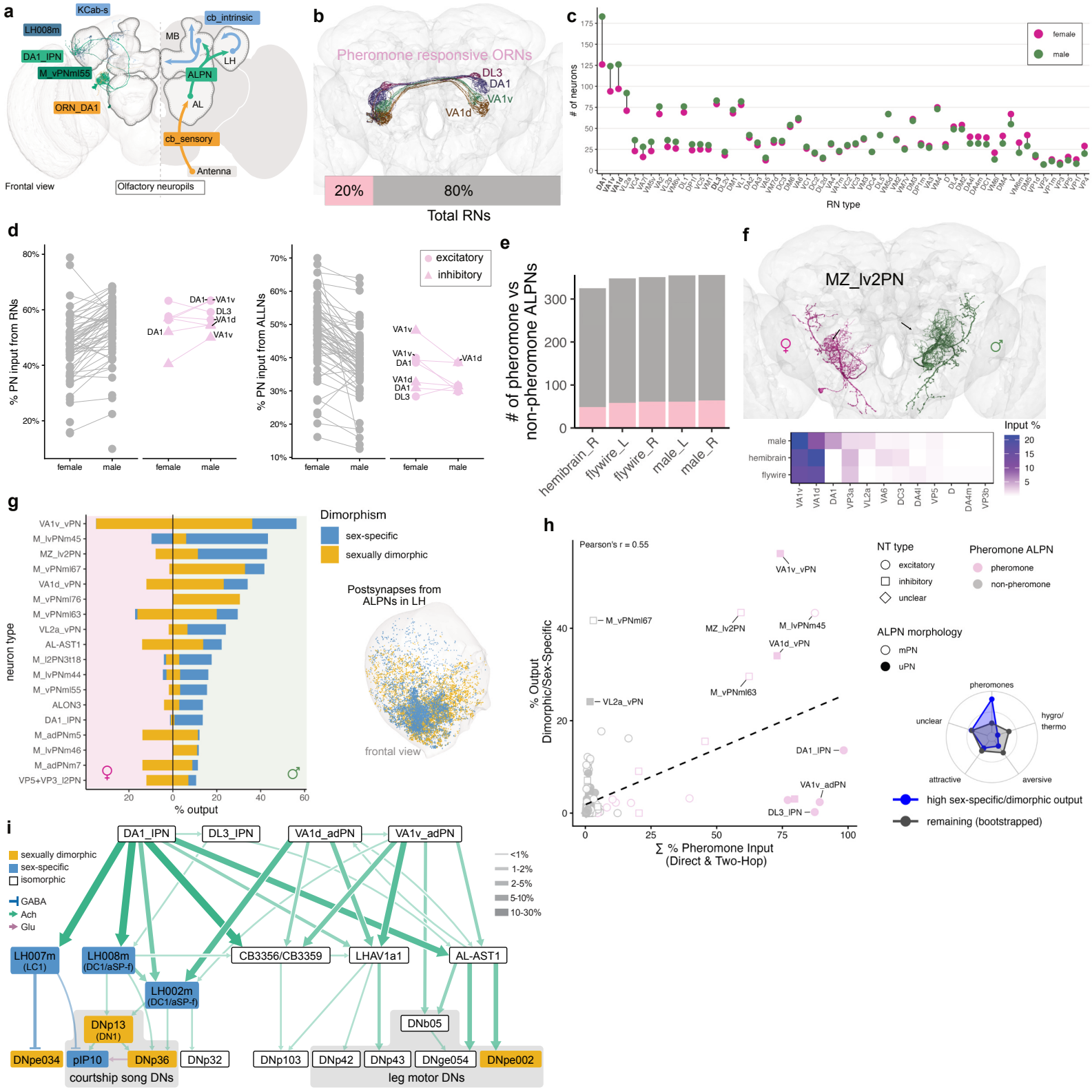

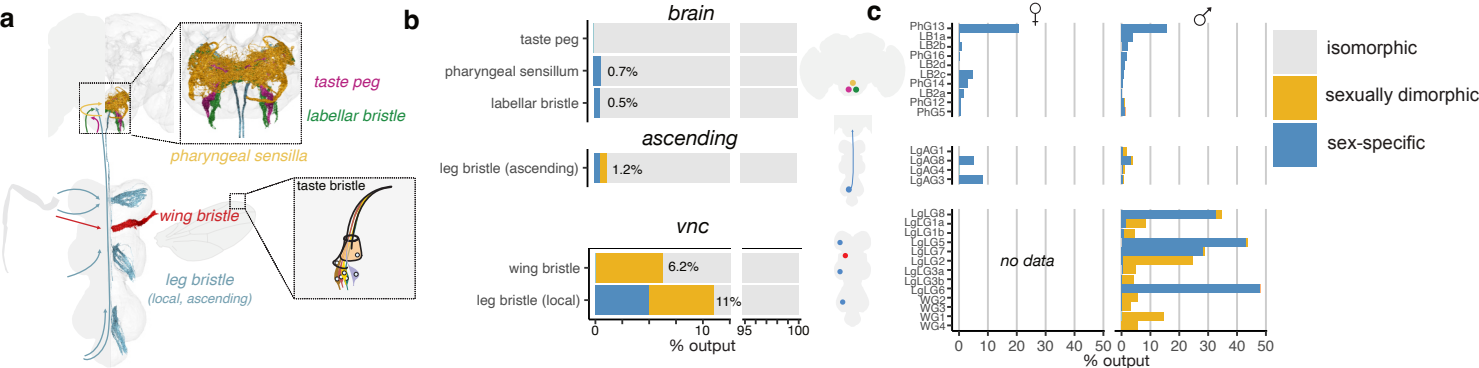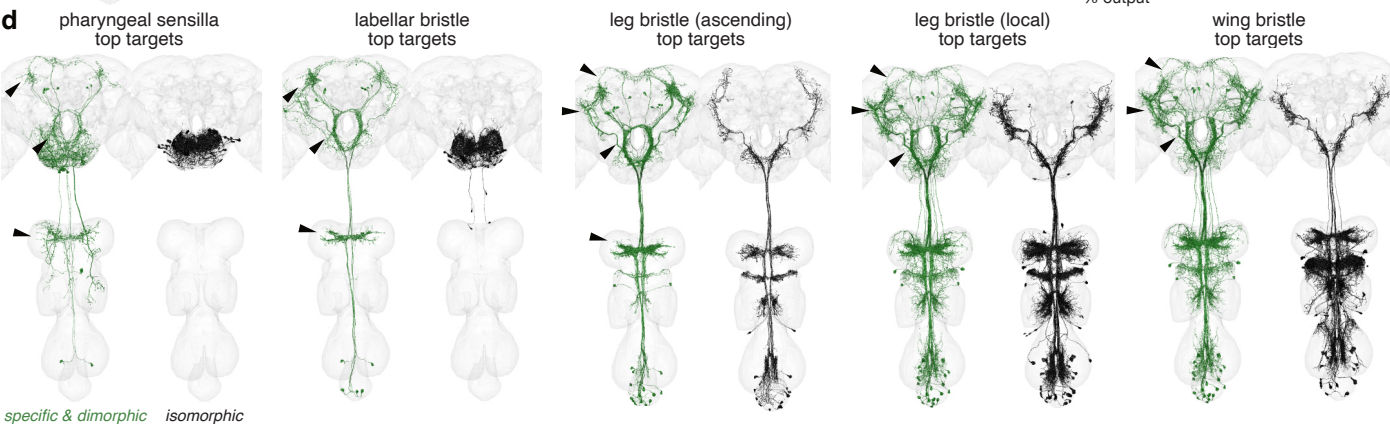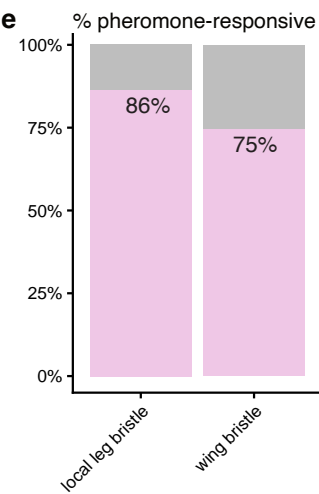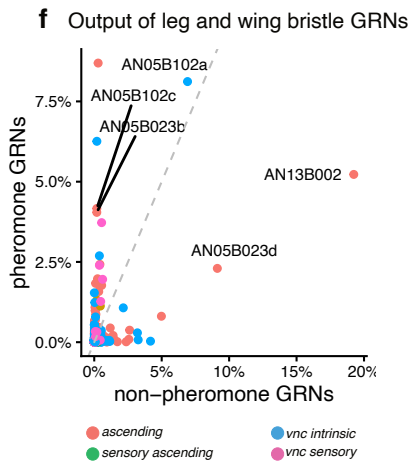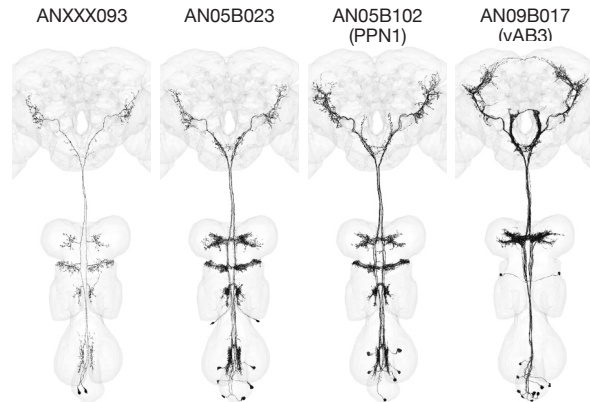

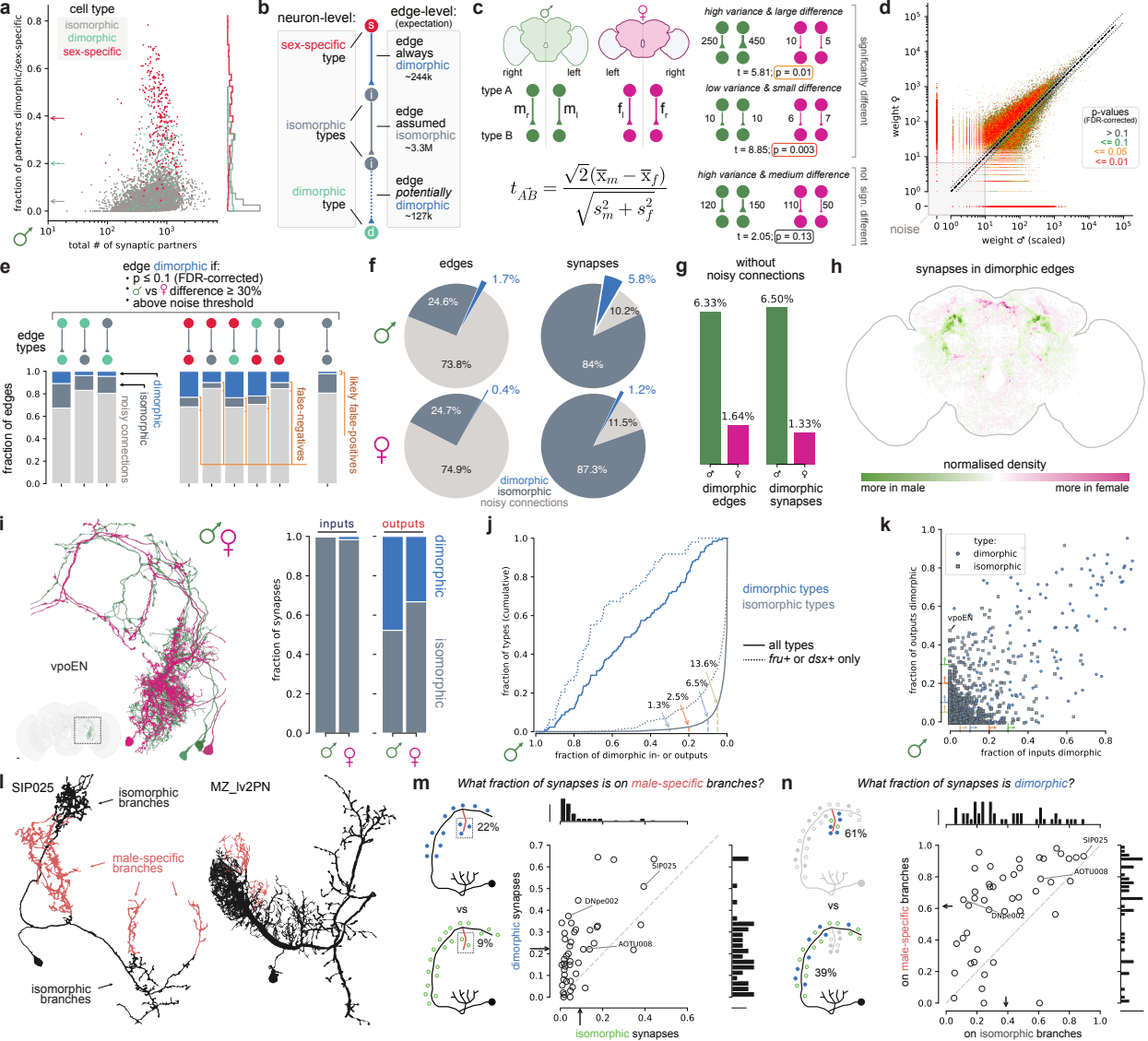

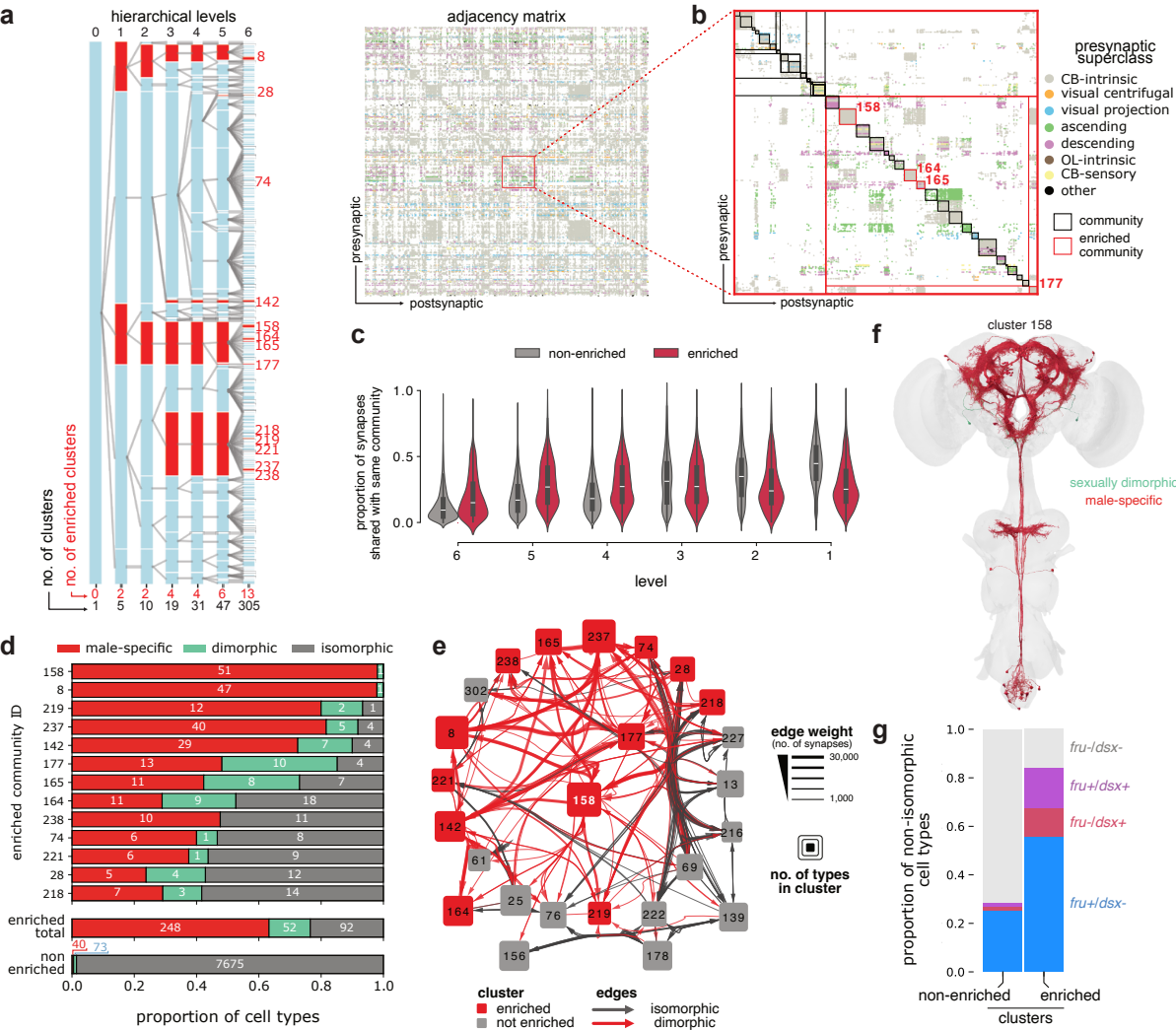

Supplement: Supplement 1 [file media-1.pdf]
