# Supplementary material for: Sexual dimorphism in the complete connectome of the Drosophila male central nervous system
Source: bioRxiv. 2025 Oct 30:2025.10.09.680999. Preprint. [Version 2] doi: 10.1101/2025.10.09.680999 (PMC12636603; doi:10.1101/2025.10.09.680999)
Supplement: Supplement 3 [file NIHPP2025.10.09.680999v2-supplement-3.pdf]

bioRxiv preprint doi: https://doi.org/10.1101/2025.10.09.680999; this version posted October 30, 2025. The copyright holder for this preprint (which was not certified by peer review) is the author/funder, who has granted bioRxiv a license to display the preprint in perpetuity. It is made available under aCC-BY 4.0 International license.

Supplementary Figures

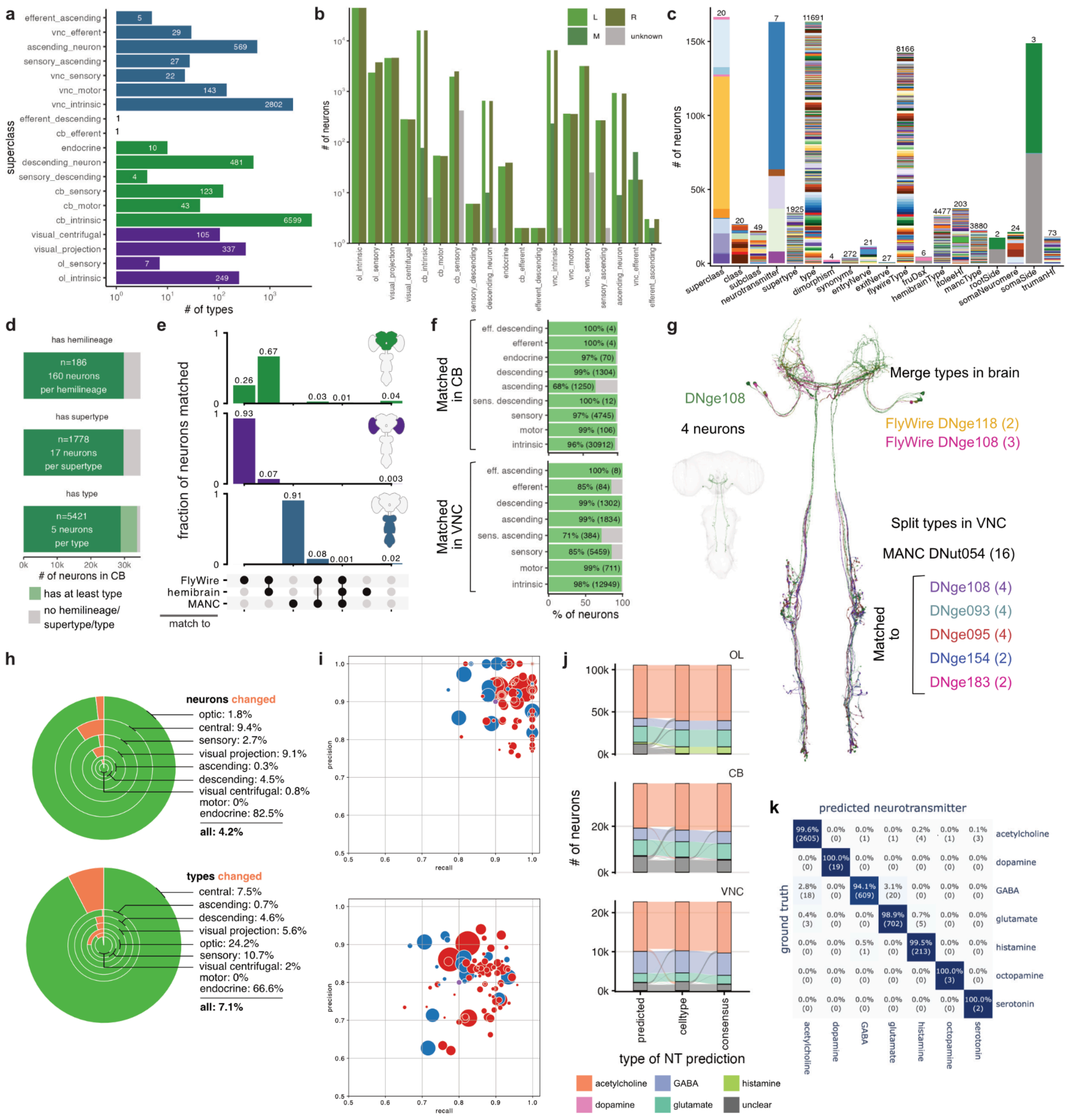

Figure S1 | Annotation, cross-matching and synapse and neurotransmitter assignment, related to figure 1 and figure S9

a Number of types per superclass and per CNS region. **b** Number of neurons by soma or root side per superclass. **c** Number of neurons with annotations for several categories.The number of unique annotations for each one is shown at the top of the bar. **d** Number of neurons in the central brain with `itoleeHl`, `supertype` and/or `type` annotations. The number of distinct annotations, and the average number of neurons per category is shown in the bar. **e** Fraction of neurons cross-matched to existing datasets per CNS region. **f** Number of cross-matched neurons in the central brain and VNC, per superclass. The number of matched neurons and the corresponding percentage is shown. **g** The descending neuron type DNge108 was matched to 2 types in FAFB/FlyWire and a subset of neurons for type DNut054 in MANC. The remaining 12 neurons in DNut054 match 4 other types in male CNS. **h** FlyWire cell types updated via cross-matching with the male CNS broken down by superclass. Top: percentage of neurons for which the type changed; bottom: percentage of

bioRxiv preprint doi: https://doi.org/10.1101/2025.10.09.680999; this version posted October 30, 2025. The copyright holder for this preprint (which was not certified by peer review) is the author/funder, who has granted bioRxiv a license to display the preprint in perpetuity. It is made available under aCC-BY 4.0 International license.

types for which at least one neuron changed. Surface area of wedges corresponds to the total number of neurons/types in that category. **i** The precision and recall of synapse T-bar predictions (top) and pre-post connections (bottom) in each region of interest (ROI). Markers are scaled by ROI size and color differentiates between brain (red) and VNC (blue) neuropils. **j** Change in neurotransmitter prediction for `predictedNt`, `celltypeNt` and `consensusNt` for each neuron in the CNS subregions. **k** Neuron-level confusion matrix for neurotransmitter prediction (`predictedNt`) on the held-out test set.

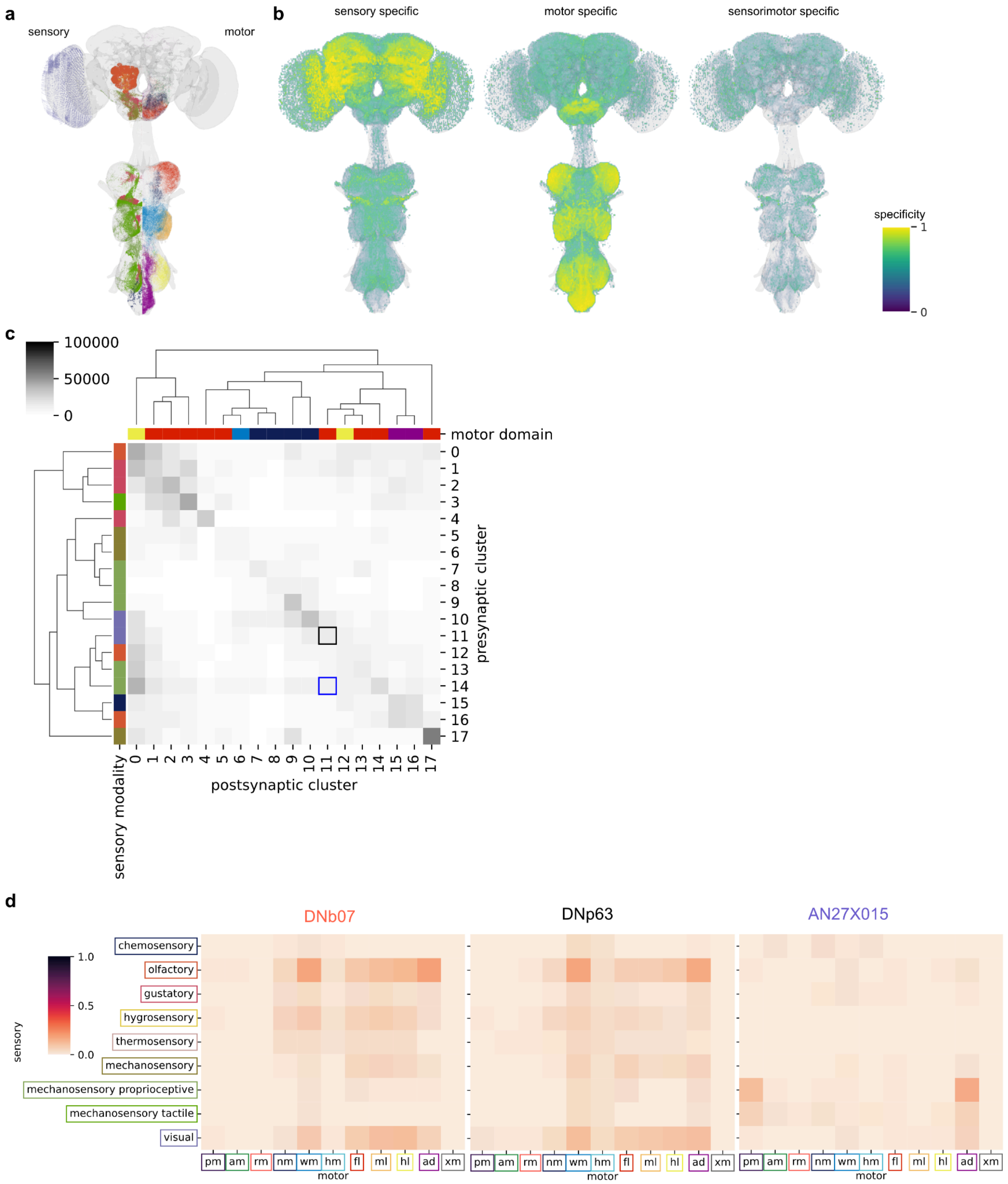

Figure S2 | related to figure 2

a Synapses from sensory (left) or motor groups (right) in the CNS. **b** Maximum intensity projection of synapses of neurons in the CNS that are specialised in flow for left: sensory group, middle: motor group, right: specific sensory-to-motor pairings. **c** Connectivity between clusters in Fig 2h clustered by

bioRxiv preprint doi: https://doi.org/10.1101/2025.10.09.680999; this version posted October 30, 2025. The copyright holder for this preprint (which was not certified by peer review) is the author/funder, who has granted bioRxiv a license to display the preprint in perpetuity. It is made available under aCC-BY 4.0 International license.

cosine similarity. Mode preferred sensory modality and motor domain are labeled. Black and blue highlights indicate the elements containing the DN-DN and AN-DN connection in Fig 2m, respectively. **d** Sensory-motor flow for the neck connective types in Fig 2m.

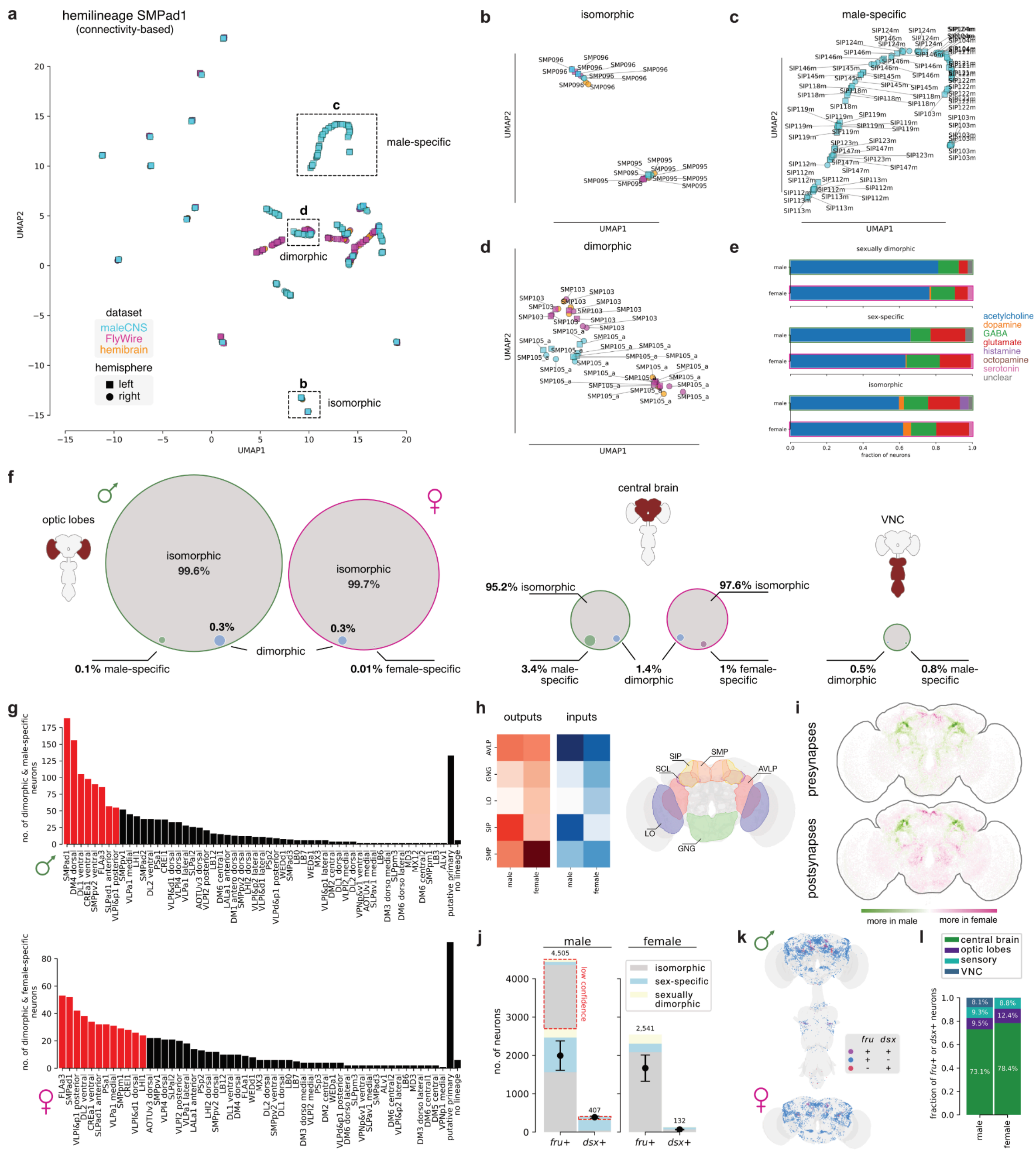

Figure S3 | Additional dimorphism analysis, related to figure 3

a UMAP embedding of connectivity-based cosine distance for neurons of the SMPad1 hemilineage from three connectomes/five hemispheres: male CNS, FlyWire and hemibrain. **b-d** Zoom-ins on insets labeled in a showing isomorphic (b), male-specific (c) and dimorphic (d) cell types. **e** Distribution of predicted neurotransmitters for dimorphic, sex-specific and isomorphic neurons. **f** Pie charts showing the fraction of dimorphic and sex-specific neurons across optic lobes, central brain and VNC. Relative sizes correspond to the number of neurons. For the VNC, our annotations in the male include 15 dimorphic types (47 neurons) from previous publications[50] that are either

bioRxiv preprint doi: https://doi.org/10.1101/2025.10.09.680999; this version posted October 30, 2025. The copyright holder for this preprint (which was not certified by peer review) is the author/funder, who has granted bioRxiv a license to display the preprint in perpetuity. It is made available under aCC-BY 4.0 International license.

VNC-intrinsic (i.e. do not have a correlate in the female brain volume), or ascending neurons for which the axonal arbors in the brain are too small to identify them in FAFB/FlyWire. Note that VNC dimorphism labeling is partial and hence represents a lower bound. **g** Number of dimorphic and sex-specific neurons per hemilineage in male (top) and female (bottom). Lineages coloured in red collectively produce ≥50% of dimorphic and sex-specific neurons. **h** Top 5 neuropil with the most dimorphic/sex-specific synapses. **i** Difference between distribution of dimorphic/sex-specific synapses in males and females. **j** Breakdown of *fruitless* (*fru*+) and *doublesex* (*dsx*+) annotations by dimorphism. This represents an upper boundary because most of the light-level data labels large populations of neurons which makes identifying individual neurons difficult. Dotted red outline indicates low-confidence annotations in the male. Pointplots in black represent mean and standard deviation of expected counts based on prior literature. **k** Cell bodies of *fru*+ and *dsx*+ neurons in male (left) and female (right). A small number of *fruitless* and *doublesex*-expressing neurons were annotated in the VNC based primarily on previous annotations from the MANC dataset[58,150].
l Distribution of *fru*+ and/or *dsx*+ neurons across main brain regions. The majority (73%) of *fruitless*/*doublesex* annotations are in the central brain, 8.1% are in the VNC, and 9.5% are in the optic lobes.

bioRxiv preprint doi: https://doi.org/10.1101/2025.10.09.680999; this version posted October 30, 2025. The copyright holder for this preprint (which was not certified by peer review) is the author/funder, who has granted bioRxiv a license to display the preprint in perpetuity. It is made available under aCC-BY 4.0 International license.

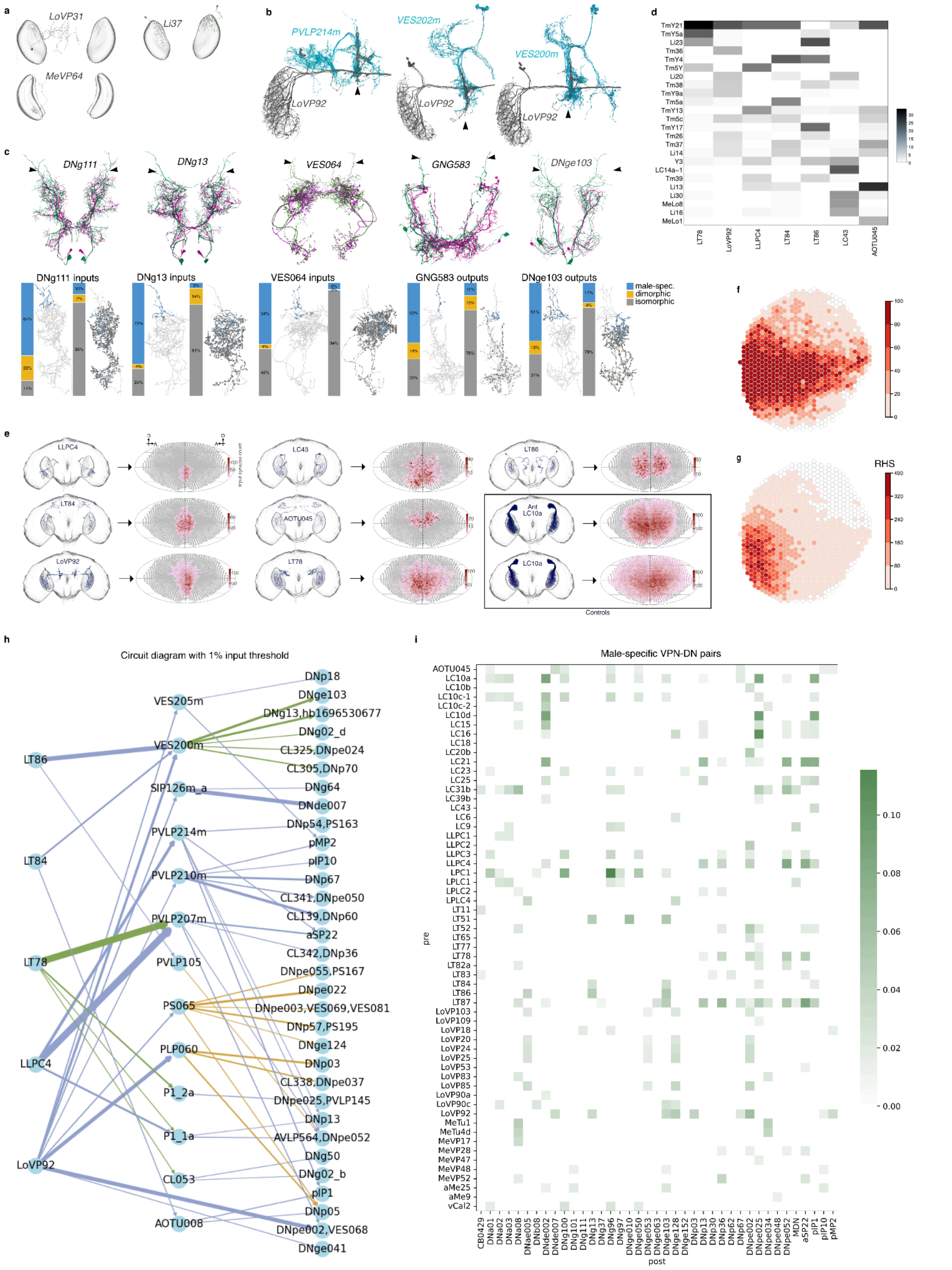

Figure S4A | Additional visual system analysis, related to figure 4

a Unmatched outlier types not considered male-specific because not left-right symmetrical. Note that Tm40 is currently classified as isomorphic. However, FAFB/FlyWire has lower cell count compared to the male CNS (41 versus 180 ids). Consequently, it was excluded from the analysis

bioRxiv preprint doi: https://doi.org/10.1101/2025.10.09.680999; this version posted October 30, 2025. The copyright holder for this preprint (which was not certified by peer review) is the author/funder, who has granted bioRxiv a license to display the preprint in perpetuity. It is made available under aCC-BY 4.0 International license.

shown in Fig 4c,l. **b** Male-specific types converging on the male-specific node defined by LoVP92 axons. **c** Dimorphic types converging on the male-specific node defined by LoVP92 axons. Stacked bar charts indicate the proportion of dimorphism status among connection partners of the dimorphic types. **d** Heatmap showing the strongest inputs from optic lobe intrinsic neurons to frontally-biased VPNs. **e** Examples of frontally-biased VPNs. Spatial coverage heatmaps show input synapse distributions mapped onto a Mollweide projection of the right compound eye's visual field. Color scale bars show input synapse count. **f** Spatial map showing all inputs to TmY21 in the ME. **g** Inputs to male-specific, dimorphic and secondary dimorphic frontal types are biased towards columns devoted to the frontal field of view. **h** All pathways from VPNs in Fig 4l, to DNs with known functions or sexual dimorphism, within two synaptic hops, where all connections are stronger than 1% input. **i** Effective connectivity based on male specific pathways, involving at least one dimorphic or sex-specific cell type, within two synaptic hops, where each connection is stronger than 1% input, from all VPNs, to DNs with known functions or sexual dimorphism. The colour is based on the sum of direct connection strength, and the square root of the products of weights in the two-hop pathways.

a

groups	# cell types R	# cell types L	# cells R	# cells L	# input conn R	# input conn L	# output conn R	# output conn L
ONIN	149	149	15.7K	14.4K	8.3M	7.3M	15.3M	15.4M
ONCN	95	95	32.4K	32.3K	13.9M	11.6M	27.6M	28.1M
VPN	352	348	4.5K	4.5K	6.0M	5.1M	9.9M	10.1M
VCN	104	104	267	273	776.1K	757.2K	2.5M	2.7M

b

neuropil	# cell types R	# cell types L	# cells R	# cells L	# input conn R	# input conn L	# output conn R	# output conn L
LA	15	22	6.9K	4.5K	395.1K	272.9K	529.7K	295.3K
ME	345	366	41.9K	41.8K	15.5M	13.3M	26.7M	27.0M
LO	422	430	25.9K	25.9K	7.4M	6.1M	14.1M	14.4M
LOP	128	125	13.1K	12.7K	3.7M	3.0M	6.0M	5.8M
AME	60	25	137	83	26.8K	7.2K	46.9K	15.1K

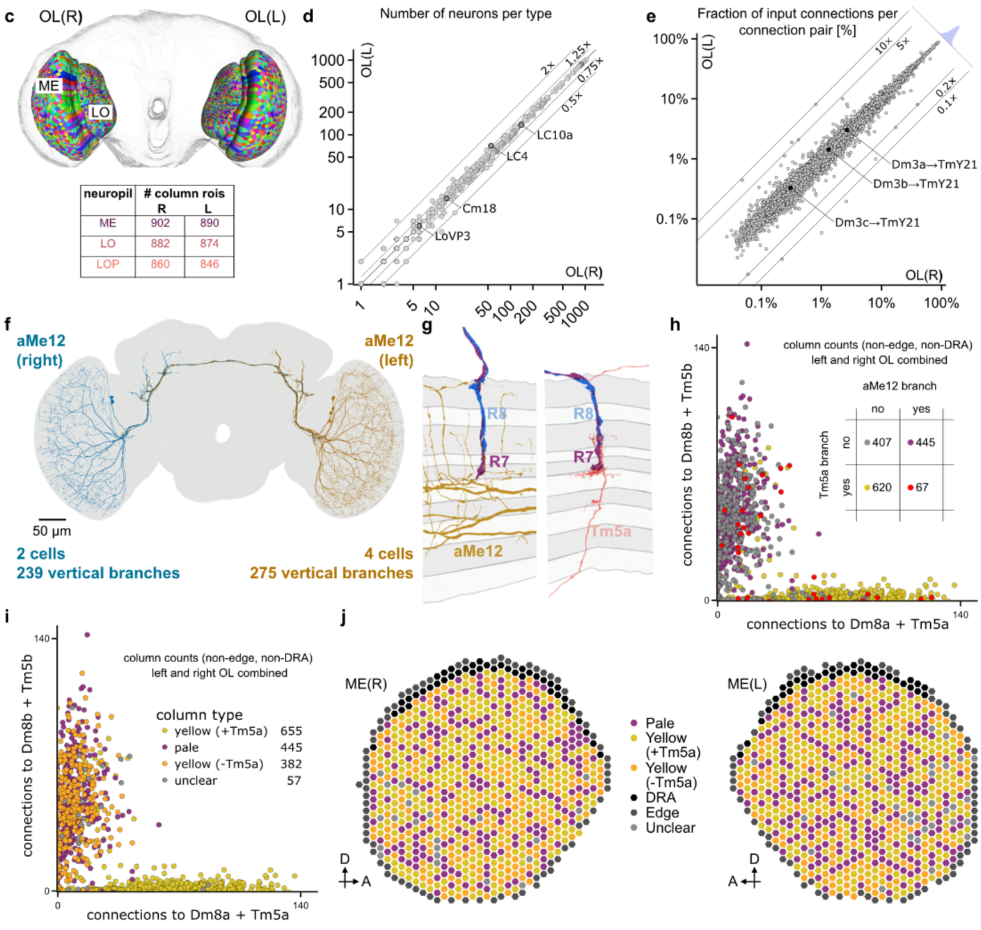

neuropil	# column rois R	# column rois L
ME	902	890
LO	882	874
LOP	860	846

bioRxiv preprint doi: https://doi.org/10.1101/2025.10.09.680999; this version posted October 30, 2025. The copyright holder for this preprint (which was not certified by peer review) is the author/funder, who has granted bioRxiv a license to display the preprint in perpetuity. It is made available under aCC-BY 4.0 International license.

Figure S4B | Complete inventory of optic lobe cell types across both brain hemispheres of the male CNS data set, related to figure 4

a Comparative summary of cell type groups showing numbers of cell types, cells, input and output connections across both optic lobes (Cell type groups follow Nern *et al.* 2025, with intrinsic neurons of the optic lobe, referred to as OLIN in Fig 4b, divided into ONIN and ONCN, Optic Neuropil Intrinsic and Connecting Neuron groups.). The table omits cell types not classified as ONIN, ONCN, VPN or VCN (the 'other' group in Nern *et al.* (2025), see Methods). Of the 701 cell types present in both hemispheres, 4 are only found on the right side, and 1 is only found on the left side. **b** The distribution of cells, cell types and connections across the optic lobe neuropils (LA: lamina; ME: medulla; LO: lobula; LOP: lobula plate; AME: accessory medulla). Some differences between left and right hemispheres appear to be due to differences in the position of neuropil boundaries between the two sides. **c** Column ROIs were updated in the right optic lobe, and developed for the left optic lobe, following the methods of Nern *et al.* (2025). This image shows a frontal view of the brain, highlighting the 'patchwork' of column ROIs across the main optic lobe neuropils. The table shows the number of column ROIs in the three columnar optic lobe neuropils. Bilateral layer ROIs are also implemented in the neuPrint database to facilitate analysis (not shown). **d** Scatter plot comparing cell counts per type between left and right optic lobes. Nearly all cell types lie near the unity line, indicating similar numbers of cells / type in both hemispheres. **e** Scatter plot comparing the number of connections per connection type (i.e. ordered pair of connected cell types), quantified as the percentage of connections relative to the total input connections of the postsynaptic type, in the left and right optic lobe. The histogram (blue), aligned with the plot, shows the distribution of ratios of left and right optic lobe input percentages. For cell types with synapses in both optic lobes, the left and right hemisphere cells were treated as different types. Connections show strong approximate bilateral symmetry across the diversity of synaptic connections between optic lobe types. The weights of the indicated connections are very similar between the two male optic lobes but differ in female flies (see Fig 4). **f** Rendering of the aMe12 neurons, whose fine arbors were systematically proofread to provide an improved anatomical marker for typing columns into pale/yellow (Kind *et al.* 2021; Nern *et al. 2025*). The number of aMe12 cells differs between the two hemispheres but the number of vertical branches (see next panel) is similar. **g** Examples of anatomical features used to type medulla columns – generally, columns with an ascending aMe12 branch (right) are typed as pale and those with a Tm5a branch (left) are typed as yellow. Columns with neither aMe12 nor Tm5a branches are also considered probable yellow columns (see below and Methods). When available, R7 photoreceptor connectivity in the column was also considered and some columns with conflicting features classified as unclear. **h** Scatter plot of R7 connections (combined for the left and right optic lobe) to the indicated neurons, with the color coding indicating the presence of Tm5a, aMe12, neither or both, by column. The scatter plot only includes columns with reconstructed R7 photoreceptors, the table inset shows counts for all columns (except in the DRA and at the medulla edge). **i** Scatter plot of R7 connections as in (h) with color-coding now indicating the different column types. Numbers included in the legend are counts for all columns (except DRA and edge). The general approach of classifying columns as pale and yellow followed Nern *et al.* (2025) but the now much more complete aMe12 reconstructions enabled a more complete typing of pale columns. Because we now expect most pale columns to be identified through aMe12 branches, we tentatively assign most of the remaining columns as yellow (yellow (-Tm5), see Methods.) **j** Medulla column maps showing left and right optic lobe column patterns with 6 column identities indicated: pale, two types of yellow columns (generally identified by the presence or absence of a vertical Tm5a branch), DRA (dorsal rim), edge columns which lack R7/R8s, and the few remaining columns indicated as 'unclear.'

bioRxiv preprint doi: https://doi.org/10.1101/2025.10.09.680999; this version posted October 30, 2025. The copyright holder for this preprint (which was not certified by peer review) is the author/funder, who has granted bioRxiv a license to display the preprint in perpetuity. It is made available under aCC-BY 4.0 International license.

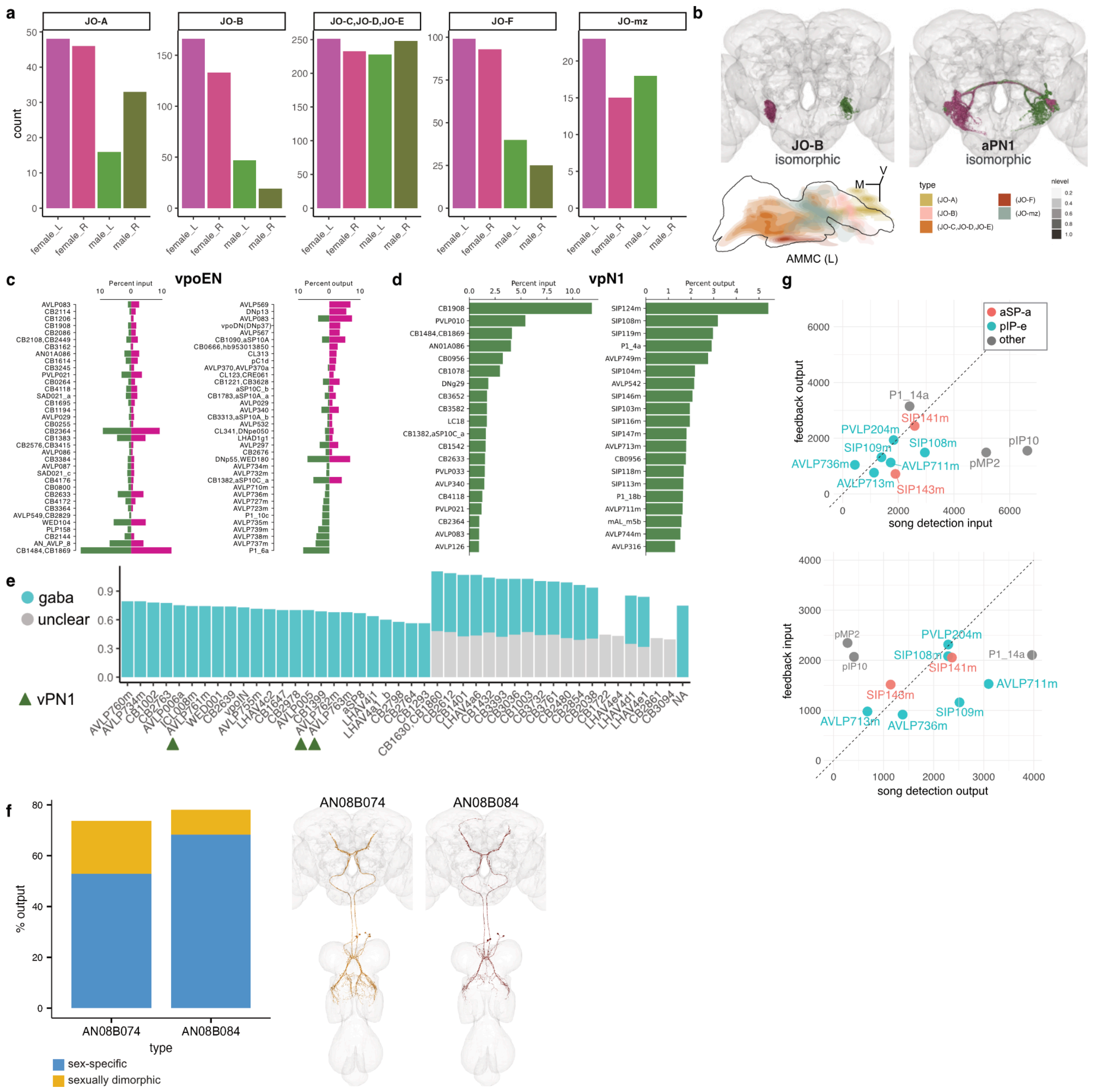

Figure S5 | Additional auditory system analysis, related to figure 5

a Johnston's Organ Neuron count by type in males (male CNS) and females (FAFB/FlyWire). Despite segmentation issues, we were able to identify around 20% of JO-B neurons. **b** Top left: JO-B neurons in female (FAFB/FlyWire, magenta) and male (male CNS, green) brains. Bottom left: Kernel distribution plots of AMMC innervation by JO neuron types in male CNS. Right: Second-order auditory neurons, aPN1, in female (FAFB/FlyWire, magenta) and male (male CNS, green) brains. **c** Top input (left) and output (right) partners of vpoEN in males (green) and females (magenta). **d** Same as in c for the male-specific vPN1 neurons (AVLP761m, AVLP762m, AVLP763m). **e** Average confidence score for consensus neurotransmitter predictions in the LHl1 lineage in male CNS; vPN1 cell types indicated by green arrowheads. **f** Left: Fraction of sexually dimorphic (gold) and sex specific (blue) outputs for AN08B074 and AN08B084. Right: AN08B074 and AN08B084 in male CNS EM space. **g** Scatter plot of the synaptic connection strength with song detection (x-axis) and AN feedback (y-axis) for cell types comprising *fruitless*-expressing clones in the circuit diagrams in Fig 5b, f.

bioRxiv preprint doi: https://doi.org/10.1101/2025.10.09.680999; this version posted October 30, 2025. The copyright holder for this preprint (which was not certified by peer review) is the author/funder, who has granted bioRxiv a license to display the preprint in perpetuity. It is made available under aCC-BY 4.0 International license.

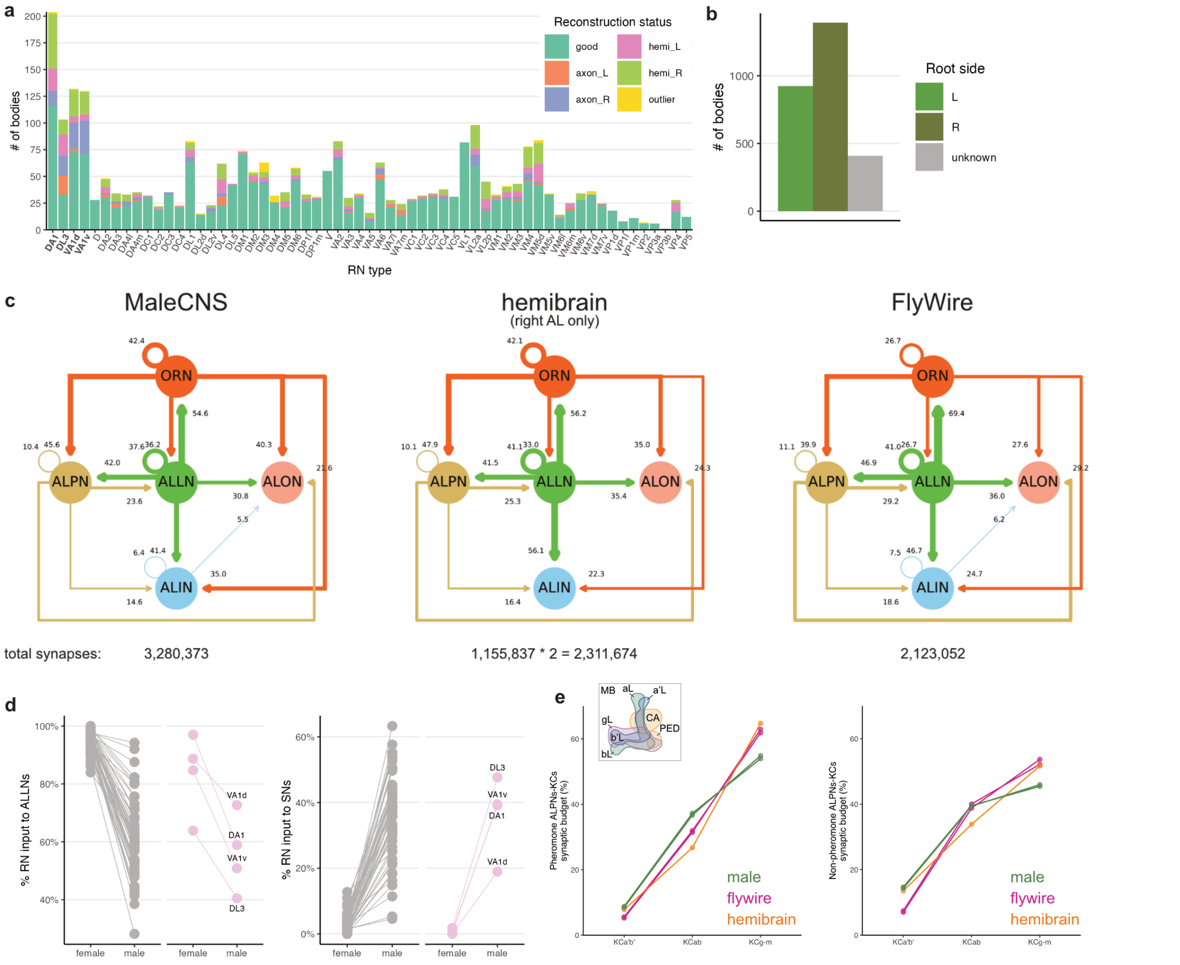

Figure S6 | Proofreading and connectivity analysis in the olfactory system, related to figure 6

a Number of bodies per RN type and its reconstruction status. axon_L/R: axon (presynapses) only present on that side, though the body crosses the midline. hemi_R/L: body ends around the midline, and only exists on one side. Outlier: cases in which a body innervates more than one glomerulus on one side or does not arborise a glomerulus proper. **b** Number of bodies per RN type and per root side. **c** Input-normalized wiring diagram between AL cell classes for AL-intrinsic connectivity in males (left) and females (FAFB/FlyWire, right). **d** Comparison of the percentage input to RNs from ALLNs (left) and sensory neurons (right), for the female (FAFB/FlyWire) and male brains. ALLNs and sensory neurons are the top 2 classes that input to RNs. **e** Comparison of the synaptic budget, as percentage for the connections from ALPNs for the main types of Kenyon cells in the male and female brains; the differences observed between datasets for the KC classes are possibly due to biological variability. Left: for pheromone ALPNs; Right: for non-pheromone ALPNs. Data for each side is shown separately for FAFB/FlyWire and the male CNS brain.

bioRxiv preprint doi: https://doi.org/10.1101/2025.10.09.680999; this version posted October 30, 2025. The copyright holder for this preprint (which was not certified by peer review) is the author/funder, who has granted bioRxiv a license to display the preprint in perpetuity. It is made available under aCC-BY 4.0 International license.

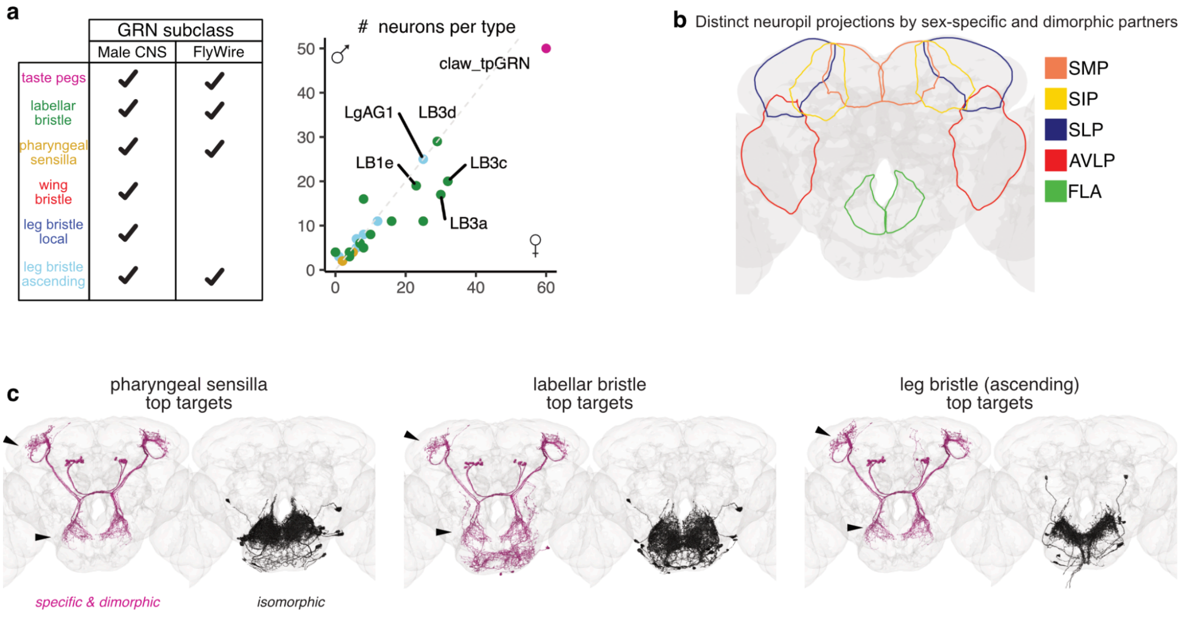

Figure S7 | Additional gustatory system analysis, related to figure 7

a Left: Table comparing male CNS subclasses of taste sensory neurons with FAFB/FlyWire data. Wing bristles and local leg bristles are exclusive to the VNC. Right: Comparison of GRN cell type counts across males and females (pink: taste pegs; green: labellar bristles; yellow: pharyngeal sensilla; red: wing bristle; navy: local leg bristles; cyan: ascending leg bristles). Note that differences in labellar bristle count appear to be technical in origin rather than true sex differences[137]. **b** Strongest neuropils innervated by second-order sex-specific and sexually dimorphic gustatory neurons. **c** Top ten targets downstream of GRN types in female (FAFB/FlyWire), by type.

bioRxiv preprint doi: https://doi.org/10.1101/2025.10.09.680999; this version posted October 30, 2025. The copyright holder for this preprint (which was not certified by peer review) is the author/funder, who has granted bioRxiv a license to display the preprint in perpetuity. It is made available under aCC-BY 4.0 International license.

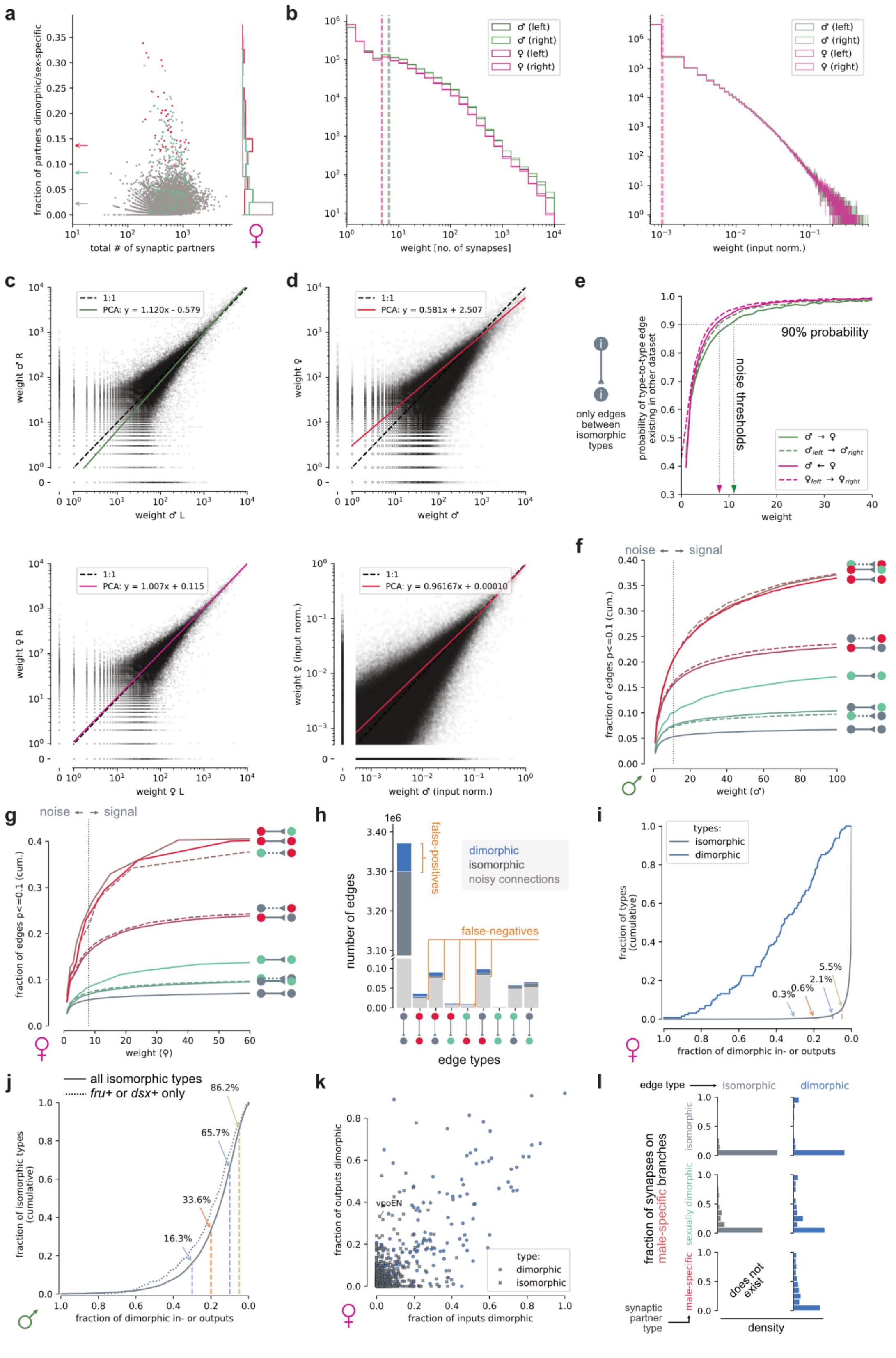

bioRxiv preprint doi: https://doi.org/10.1101/2025.10.09.680999; this version posted October 30, 2025. The copyright holder for this preprint (which was not certified by peer review) is the author/funder, who has granted bioRxiv a license to display the preprint in perpetuity. It is made available under aCC-BY 4.0 International license.

Figure S8 | Supporting dimorphism analyses, related to figure 8

a Dimorphic up- and downstream synaptic partners as a fraction of total synaptic partners for all cross-matched female cell types. Arrows on the y-axis indicate means for iso-, dimorphic and male-specific types. **b** Histogram of total (left) and input-normalised (right) cross-matched type-to-type edge weights for both male and female. Dotted vertical lines indicate the means. **c** Left versus right weights for cross-matched type-to-type edges in male (top) and female (bottom). Green/magenta line represents PCA fit. **d** Male versus female edge weights for cross-matched types. Top: total number of synapses; bottom: input normalised. Red lines represent PCA fit. **e** Probability of finding an edge between isomorphic neurons of a given weight in another hemisphere of the same or different dataset. **f** Cumulative fraction of male edges with FDR-corrected p-values below 0.1 per edge type. Dotted vertical line marks 90% probability threshold from e. **g** Same as f but in female. **h** Total number of dimorphic, isomorphic and noisy connections (male + female) broken down by edge type. **i** Fraction of iso- and dimorphic types with at least X% dimorphic in- or outputs (by synapse count) in female. **j** Fraction of isomorphic types in male with at least X% dimorphic in- or outputs (by synapse count). Same analysis as Fig 8j but using only the iso-iso edges in the graph and without removing presumed false-positive dimorphic edges. Shows that if at least one partner is fru+/dsx+, there is a slightly higher chance of edges being flagged as dimorphic. While consistent with the idea that a minority of these connections are truly dimorphic, additional connectomes would be required to make precise conclusions. **k** Fraction of dimorphic in- versus outputs (by synapse count) per cell type in the female. **l** For each edge from/to a cell type that was split into male-specific and isomorphic branches, the fraction of synapses found on male-specific branches broken down by dimorphism of the synaptic partner (rows) and edge columns).

bioRxiv preprint doi: https://doi.org/10.1101/2025.10.09.680999; this version posted October 30, 2025. The copyright holder for this preprint (which was not certified by peer review) is the author/funder, who has granted bioRxiv a license to display the preprint in perpetuity. It is made available under aCC-BY 4.0 International license.

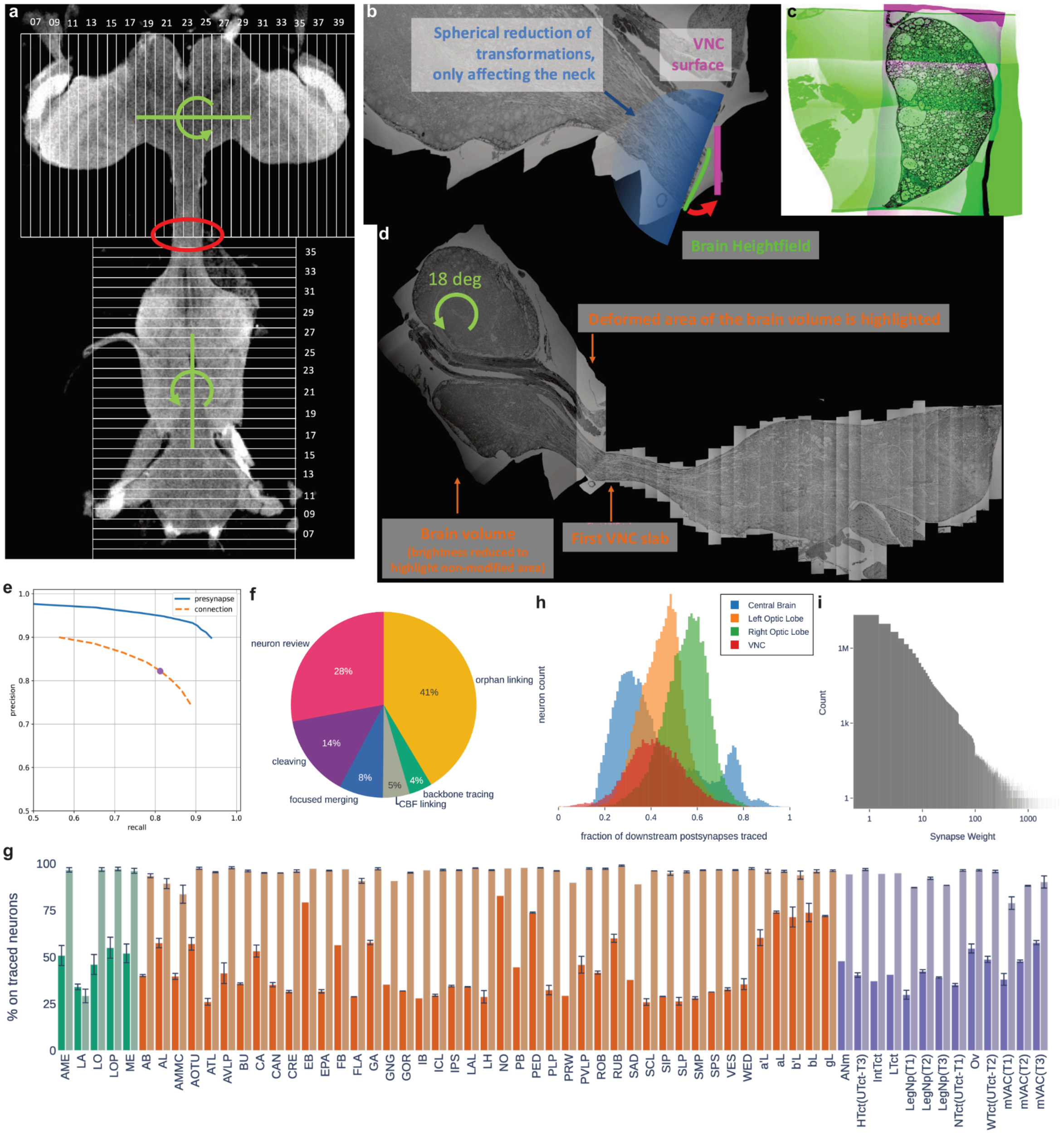

Figure S9 | Dataset alignment and proofreading metrics, related to figure 1 and Methods
a Overview of the entire CNS and the slicing into slabs for FIB-SEM imaging. The red ellipse highlights the brain/VNC cut, green rotation axes show the degrees of freedom wrt to slab orientation. **b** Transformations that were applied to the brain and VNC volumes to stitch them seamlessly. **c** Overlay of the extracted surfaces of the brain (green) and VNC (magenta). Note the 4 slabs that are visible in the brain surface. **d** Cut through the entire CNS volume highlighting the connection between brain and CNS, the modified area of the brain for stitching both volumes, and the slabs of the VNC. **e** The precision-recall curves for presynapses and connections, sweeping across the model's output confidence score. Our published synapses are filtered with a confidence threshold of 0.5 (purple dot), resulting in overall precision of 0.82 and recall of 0.81. **f** Estimated proportion of proofreading labor spent in each major proofreading protocol. **g** Traced completeness of synapses across neuropils. Light bars show presynapse completeness, dark bars show postsynapse completeness, and hue differentiates between optic lobe, central brain, and VNC neuropils. For paired left and right neuropils, the whiskers indicate the spread between left and right values and the bar shows the mean. **h** The distributions of downstream capture fraction for traced

bioRxiv preprint doi: https://doi.org/10.1101/2025.10.09.680999; this version posted October 30, 2025. The copyright holder for this preprint (which was not certified by peer review) is the author/funder, who has granted bioRxiv a license to display the preprint in perpetuity. It is made available under aCC-BY 4.0 International license.

neurons in the central brain, optic lobes, and ventral nerve cord. **i** The distribution of synaptic partner counts of remaining unmerged 'orphan' fragments.
